# Lurbinectedin in small cell lung cancer

**DOI:** 10.3389/fonc.2022.932105

**Published:** 2022-08-30

**Authors:** Anna Manzo, Vincenzo Sforza, Guido Carillio, Giuliano Palumbo, Agnese Montanino, Claudia Sandomenico, Raffaele Costanzo, Giovanna Esposito, Francesca Laudato, Edoardo Mercadante, Carmine La Manna, Paolo Muto, Giuseppe Totaro, Rossella De Cecio, Carmine Picone, Maria Carmela Piccirillo, Giacomo Pascarella, Nicola Normanno, Alessandro Morabito

**Affiliations:** ^1^ Thoracic Medical Oncology, Istituto Nazionale Tumori, “Fondazione G. Pascale” - IRCCS, Napoli, Italy; ^2^ Department of Oncology and Hematology, Azienda Ospedaliera Pugliese-Ciaccio, Catanzaro, Italy; ^3^ Oncology, San Giuseppe Moscati Hospital, Aversa, Italy; ^4^ Thoracic Surgery, Istituto Nazionale Tumori, “Fondazione G. Pascale” – IRCCS, Napoli, Italy; ^5^ Radiotherapy, Istituto Nazionale Tumori “Fondazione G. Pascale” - IRCCS, Naples –, Italy; ^6^ Pathology, Istituto Nazionale Tumori, “Fondazione G. Pascale” – IRCCS, Napoli, Italy; ^7^ Radiology, Istituto Nazionale Tumori, “Fondazione G. Pascale” – IRCCS, Napoli, Italy; ^8^ Clinical Trials Unit, Istituto Nazionale Tumori, “Fondazione G. Pascale” – IRCCS, Napoli, Italy; ^9^ Scientific Directorate, Istituto Nazionale Tumori “Fondazione G. Pascale” - IRCCS, Napoli, Italy; ^10^ Cellular Biology and Biotherapy, Istituto Nazionale Tumori, “Fondazione G. Pascale” – IRCCS, Napoli, Italy

**Keywords:** SCLC, lurbinectedin, topotecan, chemotherapy, second line

## Abstract

Few treatment options are available for patients with small cell lung cancer (SCLC) in progression after a first-line therapy. A novel therapeutic approach is represented by lurbinectedin, a synthetic derivative of trabectedin that works by inhibiting oncogenic transcription and promoting apoptosis in tumor cells. A phase II basket trial demonstrated the activity of lurbinectedin at the dose of 3.2 mg/m^2^ in patients with SCLC who had failed a previous chemotherapy, with a response rate of 35.2%, a median progression-free survival (mPFS) of 3.5 months, and a median overall survival (mOS) of 9.3 months. Common severe adverse events (grades 3–4) were hematological disorders, including anemia (9%), leukopenia (29%), neutropenia (46%), and thrombocytopenia (7%). On the basis of the positive results of this phase II study, on June 2020, lurbinectedin was approved by the Food and Drug Administration as second line for SCLC patients in progression on or after platinum-based therapy. The subsequent phase III trial comparing the combination of lurbinectedin plus doxorubicin vs. CAV (cyclophosphamide, Adriamycin, and vincristine) or topotecan did not demonstrate an improvement in overall survival, although the experimental arm showed a superior safety profile. Combinations of lurbinectedin with other drugs, cytotoxic agents and immune checkpoint inhibitors, are currently under investigation. The results of these studies should better define the optimal clinical application of lurbinectedin.

## Introduction

One of the most aggressive lung cancers is represented by small cell lung cancer (SCLC) (1), with an overall survival (OS) at 5 years of <10% and a median overall survival (mOS) of 9–11 months for patients in metastatic setting (2, 3). The most significant risk factor for developing SCLC is a history of tobacco exposure. Despite an extensive genetic characterization of SCLCs in recent years (4–7), no clear targetable alteration has emerged (6). For roughly 30 years, outcomes for patients with extensive-stage ES-SCLC have remained substantially unchanged (8–12), and only recently the combination of immune checkpoint inhibitors and standard platinum-based chemotherapy has changed the therapeutic paradigm in the first-line setting—thanks to the positive results observed in the IMpower133 and CASPIAN trials (13, 14).

In the IMpower133 trial, patients with metastatic SCLC naive for treatment were treated with atezolizumab or placebo plus carboplatin and etoposide every 3 weeks for four cycles followed by maintenance treatment with atezolizumab or placebo. This trial showed a median OS of 12.3 months (95% CI: 10.8–15.9 months) in the experimental arm vs. 10.3 months (95% CI: 9.3–11.3 months) for placebo [hazard ratio (HR) 0.70; 95% CI: 0.54–0.91; p = 0.0069) and a median progression-free survival (mPFS) of 5.2 months (95% CI: 4.4–5.6 months) for atezolizumab vs. 4.3 months (95% CI: 4.2–4.5 months) for placebo (HR 0.77; 95% CI: 0.62–0.96; p = 0.017). Benefits were consistent across patient subgroups (13). In the CASPIAN trial, patients with extensive-stage SCLC, naive for treatment, were randomized 1:1:1 to receive platinum-based chemotherapy (either carboplatin or cisplatin and etoposide) plus durvalumab, with or without tremelimumab every 3 weeks for 4 cycles followed by maintenance with durvalumab on day 1 every 4 weeks, or up to six cycles of platinum-based chemotherapy (standard arm). The combination of durvalumab and chemotherapy leads to a statistically significant improvement in OS [mOS of 12.9 months 95% CI: 11.3–14.7 months) for durvalumab plus chemotherapy vs. 10.5 months (95% CI: 9.3–11.2 months) for standard arm; HR 0.75; 95% CI: 0.62–0.91; p = 0.0032] (14). Based on the results of these two randomized trials, the first-line treatment for extensive-stage SCLC is currently platinum (carboplatin or cisplatin) plus etoposide and atezolizumab or durvalumab (15).

Unfortunately, almost all patients with metastatic disease relapse, notwithstanding high response rates (RRs) to first-line therapy. Patients with relapsed SCLCs are usually classified into platinum-sensitive, platinum-resistant, and platinum-refractory according to the treatment-free interval (TFI) (16). RRs to second-line chemotherapy are generally 20%–30% in platinum-sensitive patients (i.e., TFI >3 months) and 15% in platinum-resistant patients (i.e., TFI <3 months). Patients not responding or progressing during chemotherapy (platinum-refractory) have very poor outcomes, and further systemic therapy may not be helpful. Until 2020, topotecan, a topoisomerase 1 inhibitor, was the only second-line treatment approved for SCLC patients, with modest activity in sensitive disease. The efficacy of topotecan was evaluated in a randomized phase III clinical trial vs. the CAV (cyclophosphamide, Adriamycin, and vincristine) regimen; topotecan showed similar objective response rates (ORRs: 24.3% vs. 18.3%, p = 0.29), time to progression (13.3 vs. 12.3 weeks), and OS (25 vs. 24.7 weeks) but a better tolerability than CAV (17). The efficacy of topotecan was then evaluated in another phase III trial, in which topotecan was compared with best supportive care, showing a statistically significant prolongation of OS (25.9 vs. 13.9 weeks, p = 0.0104), better symptom control and a slower worsening of quality of life in patients with relapsed SCLC, of whom half were platinum-resistant. Adverse events (AEs) (particularly hematological) were however considerable, with 6% toxic deaths (18). Different toxicity profiles between oral and intravenous (i.v.) topotecan emerged from a subsequent phase III trial, which also demonstrated similar efficacy (19). Therefore, either oral or i.v. topotecan is recommended as second line in platinum-resistant or -sensitive relapsed SCLC, with CAV as an alternative option. Indeed, in a meta-analysis of 1,347 patients treated with topotecan, an RR of 17% was reported in patients with refractory-relapsed disease vs. 27% in those with sensitive disease (20). For sensitive relapsed SCLC, a recent randomized phase III study of second-line treatment compared the rechallenge with platinum-based chemotherapy and topotecan: roughly one-third of enrolled patients had limited disease at diagnosis (21). The rechallenge chemotherapy resulted in a longer mPFS than topotecan (4.7 vs. 2.7 months; HR 0.57; 90% CI 0.41–0.73; p = 0.0041). Therefore, rechallenge chemotherapy can be considered a reasonable alternative as second line for patients with sensitive relapsed SCLC. Overall, limited treatment options are currently available for patients with relapsed SCLC.

Lurbinectedin (PM01183) is a tetrahydropyrroloquinoline with better antitumor activity than trabectedin through the addition of a portion of tetrahydro β-carboline (22). This drug induces a specific degradation of transcribing RNA Pol II with the accumulation of DNA breaks, leading to apoptosis in tumor cells. The drug, covalently binding to CG-rich regions located within the affected gene, blocks the DNA repair mechanism, causing RNA polymerase II elongation arrest and therefore degradation (23). Furthermore, in transcriptionally dependent tumor cells such as SCLC cells, lurbinectedin could cause a separation of transcription factors from their target promoters, with the block of its transactivating activity. It may also influence the tumor microenvironment *via* suppression of tumor proliferation, matrix remodeling, angiogenesis, and immune suppression (24–26). Moreover, in mice with xenografted tumors, the combination of lurbinectedin and doxorubicin, which has a different mechanism of action and a different toxicity profile, showed a synergistic antitumor activity, supporting the rationale of the combination of these two drugs (27).

## Phase I studies

Elez et al. (28) evaluated in a phase I trial the safety and activity of lurbinectedin (PM01183) in 31 patients with advanced solid tumors (**Table 1**). The drug clearance was independent of body surface area (BSA), and a flat dose of 7 mg intravenously as a 1-h infusion every 3 weeks was recommended. The most frequent severe adverse effect was myelosuppression, occurring in 40% of patients, usually transient and manageable, never febrile. Fatigue, nausea, and vomiting were mild. A partial response was observed in a patient with pancreatic adenocarcinoma. A dose escalation study of lurbinectedin combined with doxorubicin 50 mg/m^2^ every 3 weeks was conducted by Calvo et al. (29). The starting dose of lurbinectedin was 3.5 mg [i.e., 50% of that suggested by Elez et al. (28)]. The dose escalation phase enrolled 74 patients, in whom the most common tumor type was SCLC (n = 28, 38%). Four dose levels were evaluated (3.5–5 mg). Most dose-limiting toxicities were hematological. The recommended flat dose of lurbinectedin was 4 mg in the combined regimen. Twenty-seven patients with relapsed SCLC were treated with the above therapy. Twelve patients (44%) had platinum-sensitive disease (relapse after at least 90 days) and received the protocol therapy as second line. The other 15 patients (56%) had platinum-resistant disease (time to relapse shorter than 90 days) and received the therapy as second, third, or fourth line of treatment. Median age was 62 years (range 48–73). Eight patients (29.6%) received 45 cycles of lurbinectedin alone after doxorubicin discontinuation. Grade 3 of higher toxicities comprised febrile neutropenia, fatigue, mucositis, and pneumonia. However, myelosuppression was transient and reversible for patients treated with the recommended dose of lurbinectedin. The most common adverse effects related to single-agent lurbinectedin were fatigue (n = 8, 100%), decreased appetite (50%), and alopecia (38%). ORR was 57.7%, and disease control rate (DCR) was 69.2%. As second line, ORR was 66.7% (14 of 21 patients). Moreover, ORR was 91.7% for 12 patients with platinum-sensitive disease [two complete (16.7%) and nine partial (75%) responses] and 33.3% for nine patients with platinum-resistant disease. DCR was 100% in sensitive and 55.6% in resistant disease. mPFS was 4.1 months. As second line, PFS was 4.7 months (5.8 months for sensitive and 3.5 months for resistant disease). Seven patients achieved a PFS lasting over 6 months. An expansion cohort of the above study, including SCLC patients relapsed after no more than one prior therapy, was successively treated with a lower dose of doxorubicin (40 mg/m^2^) to reduce the incidence of severe myelosuppression (30). Moreover, lurbinectedin dose has been modified at 2 mg/m^2^ of BSA based on the finding that patients with the lowest BSA had an increased risk of severe thrombocytopenia with the flat dose of lurbinectedin. On the other hand, the maximum lurbinectedin dose was capped at 4 mg for patients with BSA higher than 2 m^2^ to prevent unexpected toxicities. Twenty-eight patients were recruited in the expansion cohort: 18 (64%) had platinum-sensitive and 10 (36%) had platinum-resistant disease, including six patients with refractory tumor progressing within 30 days from platinum-based chemotherapy. Responding patients could continue to receive single-agent lurbinectedin at 4 mg/m^2^ every 3 weeks after 10 courses of combined regimen. ORR was 36% [one complete (4%) and nine partial (32%) responses], PFS was 3.3 months, and OS was 7.9 months. DCR was 72%. In the subgroup analysis, ORR 50%, PFS 5.7 months, and OS 11.5 months were recorded for patients with platinum-sensitive disease, while ORR 10%, PFS 1.3 months, and OS 4.6 months were recorded for patients with platinum-resistant disease. The main toxicity was confirmed as transient myelosuppression. Non-hematological events were mild or moderate and included fatigue, nausea, decreased appetite, vomiting, and alopecia.

**Table 1:** Clinical studies with Lurbinectedin in solid tumors and SCLC.

Study	Author	Setting	Pts	Treatment	Response rate (%)	Disease control rate (%)	Progression-free survival (months)	Overall survival (months)	Toxicity
Phase I	Elez ME, 2014	Advanced solid tumors	31	Lurbinectedin	3.6	32.6	–	–	Myelosuppression, nausea,vomiting fatigue
Phase I	Calvo E, 2017	SCLC, pretreated	27	Lurbinectedin + doxorubicin	57.7	69.2	4.1	–	Febrile neutropenia fatigue, mucositis, pneumonia
Phase I	Ponce S, 2019	SCLC, pretreated	7 12	Lurbinectedin + paclitaxel Lurbinectedin + irinotecan	71 25	71 67	4.8 5.6	- -	Grade 4 neutropenia, febrile neutropenia Grade 4 neutropenia, fatigue, nausea
Phase II	Trigo J, 2020	SCLC second-line	105	Lurbinectedin	35	68	3.5	9.3	Myelosuppression, febrile neutropenia
Phase III	Paz-Ares L, 2021	SCLC first line	613	Lurbinectedin + doxorubicin vs Topotecan or CAV	31 vs 29		4.0 vs 4.0, HR: 0.831, p=0.043	8.6 vs 7.6, HR: 0.967, p=0.703	Myelosuppression, liver toxicity

CAV: cyclophoshamide, doxorubicin, vincristine.

## Phase II studies

The evidence of lurbinectedin activity in SCLC came from a cohort of a single-arm, open-label, phase II basket trial conducted by Trigo et al. (31). The authors recruited 105 patients with advanced SCLC pretreated with only one previous line of treatment (immunotherapy was allowed, alone or in combination with chemotherapy) and an Eastern Cooperative Oncology Group (ECOG) performance status of 2 or lower. Unfortunately, the trial did not include patients with known central nervous system (CNS) involvement, missing a crucial information about the activity of lurbinectedin this setting. All patients were treated with 3.2 mg/m² lurbinectedin administered as a 1-h i.v. infusion once every 3 weeks until disease progression or unacceptable toxicity. According to the investigator assessment, after a median follow-up of 17.1 months, the study met its primary endpoint with an RR of 35.2% (95% CI: 26.2–45.2) in the entire cohort. In a preplanned analysis of overall response by treatment-free interval (TFI), the RR in the subgroup of 60 patients who had a sensitive disease (TFI of 90 days or longer) was 45.0% (95% CI: 32.1–58.4) with a median duration of response of 6.2 months (95% CI: 3.5–7.3). On the contrary, the subgroup of 45 patients with poor prognosis (TFI of less than 90 days) achieved an overall response of 22.2% (95% CI: 11.2–37.1) and median duration of response of 4.7 months (95% CI: 2.6–5.6). The mPFS was 3.5 months (95% CI: 2.6–4.3) in the study population: 4.6 months (95% CI: 2.8–6.5) in patients with a sensitive disease and 2.6 months (95% CI: 1.3–3.9) in patients with resistant disease, while the mOS was 9.3 months (95% CI: 6.3–11.8) in the overall population, 11.9 months (95% CI: 9.7–16.2) in patients with a sensitive disease, and 5.0 months (95% CI: 4.1–6.3) in patients with resistant disease. In a *post-hoc* exploratory analysis, lurbinectedin activity was observed in a small group of eight patients (8%) who had received previous immunotherapy, where five of them had durable responses according to investigator assessment. The most common grade 3–4 AEs were hematological disorders, including anemia (9%), leukopenia (29%), neutropenia (46%), and thrombocytopenia (7%). Serious treatment-related AEs were recorded in 10% of patients, principally due to neutropenia and febrile neutropenia [five (5%) patients for each]. However, no treatment-related deaths occurred. Other mild or moderate toxicities were fatigue (58%), nausea (32%), decreased appetite (21%), vomiting (18%), diarrhea (15%), and pneumonia (2%). The most common biochemical abnormalities were creatinine (83%) and transaminase (alanine aminotransferase: 72%; aspartate aminotransferase: 45%) increases. It is worth to note that 47 (45%) patients were still able to receive further antitumor treatments after lurbinectedin such as paclitaxel, carboplatin, etoposide, and topotecan. The results for the subset of patient candidates in this phase II study for a platinum rechallenge according to the National Comprehensive Cancer Network(NCCN) guidelines (TFI from the first line ≥180 days) were presented by Subbiah et al. (32). The authors reported an ORR of 60.0% (95% CI: 36.1–86.9) in the 20 patients treated with lurbinectedin with TFI ≥180 days, an median duration of response (mDoR) of 5.5 months (95% CI: 2.9–11.2), and a (DCR) of 95.0% (95% CI: 75.1–99.9). Median OS was 16.2 months (95% CI: 9.6–upper level not reached) and PFS was 4.6 months (95% CI: 2.6–7.3) after a median follow-up of 15.6 months. Of note, 60.9% and 27.1% of patients were still alive after 1 and 2 years, respectively. Taken together, these data were particularly encouraging in terms of response and survival, in comparison with historical controls, in both groups of patients with resistant and sensitive disease. Furthermore, lurbinectedin showed an acceptable safety profile, with manageable reversible myelosuppression as main toxicity. However, the absence of a control group and of patients with brain involvement represents a caveat.

Based on these positive results of a phase II study, on 15 June 2020, lurbinectedin has been approved by the Food and Drug Administration (FDA) for patients with SCLC in progression on or after platinum-based chemotherapy.

## Phase III studies

The ATLANTIS study is an open-label, randomized, multicenter phase III trial evaluating in second line the efficacy of the combination of lurbinectedin and doxorubicin compared to the investigator’s choice of chemotherapy with CAV (cyclophosphamide/doxorubicin/vincristine) or topotecan. The study enrolled pretreated patients with histologically or cytologically confirmed diagnosis of limited- or extensive-stage SCLC whose disease progressed after one prior platinum-containing line (33). Patients should have chemotherapy-free interval (CTFI; time from the last dose of first-line chemotherapy to the occurrence of progressive disease) ≥30 days and could have received prior immune checkpoint inhibitor therapy. Other inclusion criteria were adequate hematological, renal, metabolic, and hepatic function and a washout of at least 3 weeks since last prior anticancer treatment. Patients may have received whole-brain radiotherapy and prophylactic cranial irradiation or palliative radiation and concluded at least 4 and 2 weeks ago, respectively. In the trial, 613 patients were randomized to receive doxorubicin 40 mg/m^2^ on day 1, followed by lurbinectedin 2 mg/m^2^ on day 1 every 21 days or physician’s choice of CAV (cyclophosphamide 1,000 mg/m^2^ on day 1, doxorubicin 45 mg/m^2^ on day 1, and vincristine 2.0 mg total on day 1 of each 21-day cycle) or topotecan 1.5 mg/m^2^ on days 1–5 every 21 days until progression of disease, investigator decision, unacceptable toxicity, or withdrawal of consent (34).. Primary granulocyte colony-stimulating factor (G-CSF) prophylaxis has been received by all patients in all treatment arms. Stratification factors of the study were ECOG PS, CTFI, baseline brain metastasis, prior immunotherapy, and investigator’s choice between CAV and topotecan. The OS was the primary endpoint. The secondary endpoints were the difference in OS between the experimental arm and CAV, OS and PFS in patients with or without CNS involvement, PFS by independent review committee (IRC), ORR per IRC, and duration of response (DoR) per IRC. Unfortunately, the trial did not meet the primary endpoint: the difference in OS between two arms was not statistically significant, and it translated into a small improvement in OS, from 7.6 months for the control arm to 8.6 months for the experimental arm. No factors were associated with a benefit in OS based on stratification analysis. IRC mPFS was 4.0 for both arms, with an HR in favor of the combination of lurbinectedin/doxorubicin of 0.831 and a p-value of 0.043, which translated to an improvement of PFS at 6 months (31% vs. 24%) and at 12 months (10% vs. 4%). A benefit from the experimental arm was observed for patients with a longer CTFI (>180 days) and treated with immunotherapy plus chemotherapy in the first line (35). Similar RRs were reported in the two arms: 31% in the experimental arm vs. 29% in the standard arm, with a greater benefit from the experimental treatment for patients with a longer CTFI (49% vs. 29%). Moreover, the mDoR was longer in the lurbinectedin combination arm: 5.7 months vs. 3.8 months (p = 0.0012). Principal grade 3–4 AEs and laboratory abnormalities were hematological disorders, including anemia, neutropenia, febrile neutropenia, and thrombocytopenia, and they were more common in the control arm (AE grade ≥3: 75% control arm vs. 47% experimental arm), with a greater delay of the treatment in this arm (34% vs. 26%). Although this phase III trial did not meet its primary endpoint and showed comparable efficacy results in the two arms, lurbinectedin plus doxorubicin showed a superior safety and tolerability profile compared to that of the control arm with a significantly lower incidence of hematological toxicities. Moreover, the ATLANTIS trial confirmed CTFI as the most important prognostic factor for second-line SCLC treatment.

## Discussion

The positive results of the pivotal phase II study of Trigo et al. (31) led to the accelerated approval by the FDA of lurbinectedin at the dose of 3.2 mg/m^2^ for metastatic SCLC in progression after first-line chemotherapy. Lurbinectedin compared favorably to other second-line regimens in terms of activity, such as topotecan and CAV and demonstrated a better safety profile, representing a new treatment option for the second-line therapy of SCLC. However, several issues remain to be addressed: Why did lurbinectedin fail to improve OS in the ATLANTIS study? What is the activity of lurbinectedin in SCLC patients with brain metastases? What is the role of new combinations of lurbinectedin with other cytotoxic agents and immune checkpoint inhibitors? What will be the role of predictive factors?

The phase III trial comparing the efficacy of lurbinectedin plus doxorubicin vs. CAV or topotecan failed to demonstrate a better OS, although a superior safety and tolerability profile was shown by the experimental combination. A possible explanation of the negative results of the phase III trial could be the lower dose of lurbinectedin in combination with doxorubicin compared with the higher dose used in the phase II trial (2.0 mg/m^2^ vs. 3.2 mg/m^2^) that provided the maximum benefit of the drug. Unfortunately, the ATLANTIS trial did not include an experimental treatment arm of lurbinectedin as a single agent. That would have been important to confirm in a phase III trial the superiority of single-agent lurbinectedin over topotecan.

For the second question, it is unknown to date whether lurbinectedin has CNS penetration, and most of the trials with lurbinectedin have excluded patients with brain metastases. In the ATLANTIS study, patients with a history of CNS metastases were allowed and roughly 15% of patients had a baseline CNS involvement. Median OS was 4.6 and 6.6 months for patients randomized to lurbinectedin + doxorubicin and control group, respectively. Therefore, further evaluation of lurbinectedin activity in patients with CNS metastases is needed.

For the third question, new combinations of lurbinectedin with other cytotoxic agents and immune checkpoint inhibitors are currently being explored (**Table 2**). Two phase I trials evaluated the feasibility of the combination of lurbinectedin with paclitaxel or irinotecan in SCLC patients pretreated with at least one platinum-based chemotherapy (36, 37). The recommended dose of lurbinectedin was 2.2 mg/m^2^ on day 1 in combination with paclitaxel 80 mg/m^2^ on days 1 and 8 every 3 weeks. The RR in SCLC patients was 71% (67% in patients with a CTFI >90 days), with a median duration of response of 2.3 months (36). This combination was well tolerated. The most frequent toxicities were fatigue (57.1%), peripheral sensory neuropathy (57%), nausea (42.9%), and diarrhea (42.9%). The recommended dose of lurbinectedin was 2.0 mg/m^2^ on day 1 in combination with irinotecan 75 mg/m^2^ on days 1 and 8 plus G-CSF every 3 weeks (37). The RR in 12 SCLC patients was 25% (38% in patients with a CTFI >90 days), and the median duration of response was 4.6 months. Main toxicities were fatigue, gastrointestinal events, and hematological. The LUPER Trial (38) is a phase I/II trial involving SCLC patients who have progressed from a first-line chemotherapy-based treatment, with the aim to explore the feasibility and activity of the combination of lurbinectedin with pembrolizumab. In the phase I stage of the trial, patients will receive pembrolizumab plus lurbenectedin at a starting dose of 2.4 mg/m^2^, then this dose will be escalated. In the phase II stage, patients will receive pembrolizumab plus lurbinectedin at the dose found in the first phase. Primary outcome measure for phase II is ORR; estimated enrollment is for 42 patients. Results are awaited in September 2023. A similar phase I/II trial (39) is also involving SCLC patients who have progressed from a first-line chemotherapy-based treatment. In the phase I stage of the trial, patients will receive ipilimumab and nivolumab plus lurbenectedin at three different doses (1.5, 2.6, and 3.2 mg/m^2^). In the phase II stage, patients will receive ipilimumab and nivolumab plus lurbinectedin at the recommended dose found in the first phase. The primary outcome measure for phase II is DCR; estimated enrollment is for 57 participants. Results are awaited in October 2025. Another phase I/II trial (40) is enrolling patients with SCLC and high-grade neuroendocrine tumors who have failed to respond to previous standard treatments. Patients will receive a combination of lurbinectedin and berzosertib, an ataxia telangiectasia and Rad3-related (ATR) protein kinase inhibitor. The inhibition of ATR is cytotoxic in SCLC, and berzosertib has been found to be active in combination with topotecan in this clinical setting (41). Primary outcome measure is clinical RR, calculated as the fraction of patients who will experience a partial response (PR) or a complete response(CR); estimated enrollment is for 75 participants; study completion date is awaited in December 2026. The IMforte trial (42) is a phase III trial designed for patients with ES-SCLC who have already received a first-line induction therapy with carboplatin, etoposide, and atezolizumab and are found to have at least a stable disease or ongoing response. In arm A, patients will receive the combination of atezolizumab and lurbinectedin, while in arm B, patients will receive standard maintenance therapy with atezolizumab. PFS and OS are the primary outcome measures. Estimated enrollment is for 690 participants. Results are awaited in March 2025. The EMERGE 402 trial (43) is a phase IV trial that aims to report the efficacy and the AEs tied to lurbinectedin in the second-line ES-SCLC setting. ORR is the primary outcome measure. Estimated enrollment is for 300 participants. Results are awaited in June 2024.

**Table 2:** Ongoing studies with Lurbinectedin.

Trial	Phase	Diagnosis	Line of treatment	Pts	Treatment	Endpoints
LUPER NCT04358237	I/II	SCLC	Second-line	42	Lurbinectedin + pembrolizumab	ORR
NCT04610658	I/II	SCLC	Second-line	57	Ipilimumab + nivolumab + lurbenectedin	DCR
NCT04802174	I/II	SCLC and High Grade Neuroendocrine tumors	Advanced	75	Lurbinectedin and berzosertib	ORR
IMFORTE NCT05091567	III	SCLC not progressed after carboplatin, etoposide and atezolizumab	Maintenance	690	Lurbinectedin + atezolizumab vs atezolizumab	PFS, OS
Emerge 402 NCT04894591	IV	SCLC	Second-line	300	Lurbinectedin	ORR

Pts, patients; ORR, objective response rate; DCR, disease control rate; PFS, progression-free survival; OS, overall survival.

For the fourth question, we currently do not have biomarkers to identify SCLC patients responding to lurbinectedin or to other agents. However, Schlafen-11 (SLFN11), a predictive biomarker of response to cisplatin and to other DNA-damaging agents such as poly ADP ribose polymerase (PARP) inhibitors in multiple cancer types including SCLC, has been recently identified as a promising predictive biomarker of response also to lurbinectedin. An *in vitro* and *in vivo* study showed that cell lines with a high expression of SLFN11 protein were more sensitive to single-agent lurbinectedin (44). Moreover, in SLFN11-low SCLC cell lines that are resistant to lurbinectedin, the addition of ceralasertib, an ATR inhibitor, resensitized resistant cells, providing a rationale for combining lurbinectedin with ATR inhibitors to overcome resistance in SCLC with low SLFN11 expression. Therefore, SLFN11 immunohistochemistry (IHC) could be translated into the clinical setting and be used in clinical studies with lurbinectedin in SCLC. Moreover, the recent identification by Rudin et al. (45) of four different molecular subtypes of SCLC defined by differential expression of four key transcription regulators highlights the heterogeneity of SCLC and could allow a better customization of treatments.

In conclusion, lurbinectedin has demonstrated significant activity as a single agent in second-line therapy of SCLC, especially in platinum-sensitive patients, but failed to demonstrate an improvement in OS when combined with doxorubicin compared with CAV or topotecan. New combinations of lurbinectedin with other cytotoxic drugs and with immune checkpoint inhibitors are currently under investigation. The results of these studies should better define the optimal clinical application of lurbinectedin in SCLC.

## Author contributions

Conceptualization, AnM and AlM; writing—original draft preparation, AnM, VS, GC, GP, and AlM; writing—review and editing, AnM, VS, GC, GiuP, AgM, RC, GE, FL, EM, CM, PM, GT, RDC, CP, MP, GiuP, NN, and AlM; supervision, AlM; All authors have read and agreed to the published version of the manuscript.

## Acknowledgments

The Authors are grateful to Dr. Alessandra Trocino, Librarian at IRCCS “G. Pascale” of Naples, Italy, for the bibliographic assistance.

## Conflict of interest

AlM declares the following conflicts of interest: Speaker’s fee or advisory board: MSD, BMS, Boehringer, Pfizer, Roche, AstraZeneca, Novartis, Takeda, Eli-Lilly. NN declares the following personal financial interests (speaker’s fee and/or advisory boards): MSD, Qiagen, Bayer, Biocartis, Incyte, Roche, BMS, MERCK, Thermofisher, Boehringer Ingelheim, Astrazeneca, Sanofi, Eli Lilly; Institutional financial interests (financial support to research projets):MERCK, Sysmex, Thermofisher, QIAGEN, Roche, Astrazeneca, Biocartis. Non-financial interests:President, International Quality Network for Pathology (IQN Path); President, Italian Cancer Society (SIC).

The remaining authors declare that the research was conducted in the absence of any commercial or financial relationships that could be construed as a potential conflict of interest.

## Publisher’s note

All claims expressed in this article are solely those of the authors and do not necessarily represent those of their affiliated organizations, or those of the publisher, the editors and the reviewers. Any product that may be evaluated in this article, or claim that may be made by its manufacturer, is not guaranteed or endorsed by the publisher.
